# Determinants of adolescent reproductive health service utilization by senior high school students in Makassar, Indonesia

**DOI:** 10.1186/s12889-019-6587-6

**Published:** 2019-03-11

**Authors:** Fajrin Violita, Ella Nurlaela Hadi

**Affiliations:** Department of Health Education & Behavioral Sciences, Faculty of Public Health Universitas Indonesia, D Building Kampus Baru UI, Depok, 16424 Indonesia

**Keywords:** Utilization, Reproductive Health, Adolescents, Makassar

## Abstract

**Background:**

Adolescents face many problems due to risky behavior. As a result, they require special consideration through the administration of health education and reproductive health services. However the utilization of adolescent reproductive health service programs in Makassar is still relatively low. The purpose of this study, then, was to identify the rates at which adolescent reproductive health services are utilized and to analyze the determinants affecting such utilization.

**Method:**

This research was a quantitative project with a cross-sectional design, and it was conducted in March to May of 2018 in Makassar City. Data were collected via the independent completion of questionnaires by 383 senior high school students randomly selected from a total of six schools. Data were analyzed using chi-square testing and multiple logistic regression using SPSS.

**Results:**

This study found that only 24.3% of the students took advantage of adolescent reproductive health services. The results of the analysis proved that knowledge of reproductive health and available services (OR = 1.74; 95% CI = 1.040–2.911) are related to the utilization of those services. It was found that students with high levels of knowledge are nearly twice as likely to utilize adolescent reproductive health services as those with low levels of knowledge after the results were controlled for the variables of family and peer support.

**Conclusion:**

It is necessary to promote socialization between students and parents on a regular basis, disseminate information through online media/social networking, administer peer educator training, and establish school organizations in the field of reproductive health to increase awareness and utilization of adolescent reproductive health services.

## Background

Approximately 16.8% of the world’s population are adolescents, with as many as 80% of those are from developing countries [1]. In 2016, according to that year’s National Labor.

Force Survey (Sakernas), 62.89% of Indonesian adolescents aged 15–19 years were still in school. In fact, population projections indicate the possibility of a demographic surge in 2030, at which time these adolescents will be at a reproductive age [2].

Adolescents as a state asset have become a group requiring special attention. Certain health issues among adolescents are often ignored, such as those involving reproductive health, HIV-AIDS, and maternal death, the leading causes of morbidity and mortality among adolescents to date [3].

In Indonesia, risky adolescent behavior begins with dating from the ages of 15–19. The rate is quite high at 33.3% for girls and 34.5% for boys [4]. In 2015, it was reported that 8.26% of adolescent boys in the cohort, and 4.17% of girls had engaged in premarital sex [5]. Premarital sex can lead to an increased risk of contracting sexually transmitted diseases, the most devastating of which is usually HIV-AIDS. In fact, the proportion of HIV infection among those aged 15–24 years continues to increase in Indonesia, from 18.4% in 2014, to 19.3% in 2015, and 21.0% in 2016 [6].

Efforts to overcome adolescent reproductive health issues have been endorsed since the Adolescent Friendly Reproductive Health Service (AFRHS) program was established in 1994 at the International Conference on Population and Development (ICPD) meeting in Cairo [7]. In Indonesia, version of the program has been in place since 2003 under the name of Adolescent Care Health Service (PKPR). In addition to PKPR, National Family Planning Coordinating Board (BKKBN) has also established a risky behavior prevention program for adolescents through an organization called the Adolescent/Student Information and Counseling Center (PIK R/M). The group trains adolescents to act as peer educators [4].

However, the utilization of adolescent reproductive health services tends to be low. Research in Ethiopia has found that as many as 62.8% of adolescents aged 15–24 years had never utilized adolescent reproductive health services [1]. Such is also the case in Indonesia. Sitorus reports that in Bali, 62.0% of adolescents have never utilized PKPR [8], and Wulandari, in Tanjung Balai City, North Sumatra, found that 53.5% of students have never taken advantage of PIK-Adolescent services [9].

Makassar City has six Community Health Center implementers for its Adolescent Care Health Service/PKPR, and 62 schools with Information and Adolescent Counseling Center/PIK-R facilities [10, 11]. Yet, the utilization of reproductive health services is low, based on the results of a December 2017 preliminary study with senior high school students in Makassar City. The same preliminary study also found that only 48.6% of the participating students used PIK-R services, while none of them had ever utilized PKPR’s services.

The low utilization rates of reproductive health services by adolescents is influenced by many factors. Several studies have found that knowledge [12, 13]; individual perceptions such as susceptibility, severity, and seriousness [13, 14]; perceived benefits and barriers [13, 15]; and support of family and peers [16, 17] have an effect on utilization rates. These factors are components of the Health Belief Model (HBM) that is often used to determine why people do oe do not participate in health programs [18]. Identifying factors affecting the health service use is important for the improvement of services. Therefore, the purpose of this study was to a) assess utilization rates of adolescent reproductive health services by senior high school students in Makassar City, and b) analyze the determinants affecting such utilization.

## Method

This was a quantitative study with a cross-sectional design conducted from March to May of 2018 in Makassar City. The population consisted of senior high school students who were located in the sub-districts of the targeted area of community health centers which implementing the PKPR and having PIK-R. Randomly selected schools from each sub-district included SMAN 9 in the Rappocini sub-district, SMAN 2 in the Mamajang sub-district, SMAN 17 in the Tallo sub-district, SMKN 1 in the Tamalate sub-district, SMA Muhammadiyah 6 in the Wajo sub-district, and SMKN 7 in the Ujung Pandang sub-district.

The minimal sample group of 372 subjects was calculated by using a two-proportion hypothesis test and randomly chosen from the selected schools. The inclusion criteria were that they be active students and willing respondents. Students in the first and third grades were excluded from this study due to the fact that for first grade students, their time spent in school had not yet reached 12 months, while the measurement for utilization of adolescent reproductive health services was based on participation or use of service during the last 12 months. For third grade students, exclusion was a result of intense preparation requirements for the national exam.

The data were provided independently by the respondents (self-reported) using a questionnaire that was tested for validity and reliability with 34 students from MAN 2 Model Makassar. It consisted of eight points of inquiry: (1) respondents’ demographic data, (2) independent variables including knowledge of reproductive health issues and available services, (2) perceived susceptibility to adolescent reproductive health issues, (3) perceived severity and seriousness of adolescent reproductive health issues, (4) perceived benefits from and (5) perceived barriers for utilizing such services, (6) family support, and (7) peer support, as well as (8) the identification of dependent variable such as the utilization of adolescent reproductive health services. The questionnaire was developed by modifying a WHO [19] questionnaire in terms of the knowledge variable and utilization of adolescent reproductive health services. The Champion’s HBM Scale [20] questionnaire was modified. Categorization of independent variables used the median value as a cut-off point, since the could not be precisely distributed. The data collected were then analyzed by using chi-square testing and multiple logistic regression with SPSS software.

Prior to data collection in the field, the study was approved by the Health Research Ethics Committee of the Faculty of Public Health University of Indonesia (Certificate Number 75/UN2.F10/PPM.00.02/2018).

## Results

Makassar City has 46 community health centers spread over 14 sub-districts, but the implementation of PKPR as of 2017 was reported only in six community health centers. Those were Kassi-Kassi, Cendrawasih, Jumpandang Baru, Jongaya, Andalas, and Makassau Community Health Centers. The 383 student respondents were from six senior high schools in the work area of the PKPR community health centers with PIK-Adolescents. The majority (81.5%) were 16–17 years old with an average age of 16.48 years, 68.1% were female, and the majority (89.3%) were Muslim (Table 1).

**Table 1:** Characteristics of respondents

General Characteristic	Total	Percentage
	N	%
Age		
15 years old	34	8.9
16 years old	168	43.9
17 years old	144	37.6
18 years old	37	9.7
Average	16.48	
Sex		
Female	261	68.1
Male	122	31.9
Religion		
Muslim	342	89.3
Protestant	30	7.8
Catholic	9	2.3
Hindu	2	0.5

The utilization of adolescent reproductive health services was first measured by asking subjects about their use of service components such as education, consultations, examinations, and treatments during the last 12 months. Based on the analyzed results, the utilization of the services by senior high school students in Makassar was still relatively low at 24.3% (Table 2).

**Table 2:** Distribution of independent variables

Variable	Total	Percentage
	N	%
Utilization		
Yes	93	24.3
No	290	75.7
Knowledge		
High	226	59.0
Low	157	41.0
Perceived Susceptibility		
High	192	50.1
Low	191	49.9
Perceived Severity and Seriousness		
High	200	52.2
Low	183	47.8
Perceived Benefit		
High	315	82.2
Low	68	17.8
Perceived Barriers		
High	166	43.3
Low	217	56.7
Family Support		
Enough	265	69.2
Less	118	30.8
Peer Support		
Available	215	56.1
Unavailable	168	43.9

Controlling for independent variability, about half (59.0%) of the students had high levels of knowledge about reproductive health and the services available. Based on the variable of individual perception, 50.1% of students had high rates of perceived susceptibility to reproductive health issues, 52.2% had high rates of perceived severity and seriousness of reproductive health issues, 82.2% had high rates of perceived benefits, and 43.3% had low rates of perceived barriers to utilizing the adolescent reproductive health services. Meanwhile, with respect to the variables of family and peer support, 69.2 and 56.1% of the students reportedly had sufficient levels (Table 2).

In Table 3, bivariate analysis showed that knowledge of reproductive health and available services (*p* = 0.010; OR = 1.986; 95% CI = 1.200–3.288), family support (*p* = 0.018; OR = 2.019; 95% CI = 1.154–3.352) and peer support (*p* = 0.007; OR = 2.032; 95% CI = 1.238–3.335) were both (1) factors related to the utilization of adolescent reproductive health services and (2) candidates entering into multivariate modeling. By contrast, the variables of individual perception (perceived susceptibility to adolescent reproductive health issues, perceived severity and seriousness of adolescent reproduction, and barriers to using services) were insignificantly associated with the utilization of health services (*p*-value > 0, 05).

**Table 3:** Determinants associated with the utilization of adolescent reproductive health services by senior high school students in Makassar City

Independent Variable	Utilization of Adolescent Reproductive Health Services				Total		OR (CI = 95%)	*p-value*
	Yes		No					
	n	%	n	%	n	%		
Knowledge								
High	66	29.2	160	70.8	226	100	1.986 (1.200–3.288)	0.010*
Low	27	17.2	130	82.8	157	100		
Perceived Susceptibility								
High	46	24.0	146	76.0	192	100	0.965 (0.605–1.540)	0.977
Low	47	24.6	144	75.4	191	100		
Perceived Severity and Seriousness								
High	53	26.5	147	73.5	200	100	1.289 (0.805–2.064)	0.348
Low	40	21.9	143	78,1	183	100		
Perceived Benefit								
High	76	24.1	239	75.9	315	100	0.954 (0.502–1.750)	1.000
Low	17	25.0	51	75.0	68	100		
Perceived Barriers								
Low	38	22.9	128	77.1	166	100	0.874 (0.544–1.405)	0.664
High	55	25.3	162	74.7	217	100		
Family Support								
Enough	74	27.9	191	72.1	265	100	2.019 (1.154–3.352)	0.018*
Less	19	16.1	99	83.9	118	100		
Peer Support								
Available	64	29.8	151	70.2	215	100	2.032 (1.238–3.335)	0.007*
Unavailable	29	17.3	139	82.7	168	100		

*Related to the utilization of adolescent reproductive health services and candidates into multivariate modelling

Multiple logistic regression analysis demonstrated that knowledge of reproductive health issues and available services (*p* = 0.035; OR = 1740; 95% CI = 1.040–2.911) was a significant factor related to the utilization of adolescent reproductive health services, while the variables of family and peer support showed confounding variables. Students with high levels of knowledge about reproductive health and available services were nearly twice as likely to utilize adolescent reproductive health services as compared to those with low levels knowledge after the results were controlled for family and peer support (Table 4).

**Table 4:** Multivariate result

Variable	B	*p-value*	OR	CI (95%)
Knowledge	0.554	0.035	1.740	1.040–2.911
Family Support	0.456	0.129	1.577	0.875–2.843
Peer Support	0.613	0.055	1.664	0.988–2.802

## Discussion

The utilization of adolescent reproductive health services by senior high school students in Makassar City was still low (24.3%). In fact, the result was lower than those in the Rokan Hulu district, Pekanbaru and the Gianyar district, Bali, which reported that 33.6% of adolescents had used PIK-Adolescent reproductive health services in schools and 38% had utilized PKPR, respectively [9, 17]. Meanwhile, research in Kenya, Africa conducted in 2017 reported similar findings, with only 38.5% of adolescents having utilized adolescent reproductive health services [21].

Looking more closely at the utilization of reproductive health services, most students (63.4%) utilized the services once in the last 12 months, with NAPZA and cigarettes education being the most widely-used type of service (52.7%). The most frequently visited service provider was PKPR (46.2%), and doctors/ and/ or nurses were the most common service providers seen (61.3%). Regarding the quality of service, the majority of students stated that the service was good enough and that there was good material satisfaction (80.6%), friendly service providers (93.5%), sufficient numbers of personnel (74.2%), appropriate and comfortable service times (61.3%), and guaranteed confidentiality throughout (64.5%) (Table 5).

**Table 5:** Distribution of frequency, type and quality of services used

Frequency, Type and Quality of Services Used	Total	Percentage
	N	%
Frequency of Utilization Unavailable 1x	59	63.4
NAPZA & Cigarettes Education as the most used services	49	52.7
PKPR as the most frequently visited provider	43	46.2
Doctor/Nurses as the most frequently met	57	61.3
Quality – Good Material	75	80.6
Quality – Friendly Providers	87	93.5
Quality – Sufficient numbers of personnel	69	74.2
Quality – Appropriate Service Times	57	61.3
Quality – Guaranteed Confidentiality	60	64.5

Knowledge is one of the driving factors in behavioral change. Several studies have shown that adolescents with good knowledge of reproductive health will benefit from available services [12, 22, 23]. These studies generally reported similar results: knowledge of reproductive health and available services is a factor related to the utilization of adolescent services. Indeed, students with high levels of knowledge of reproductive health and available services were nearly two times more likely to utilize adolescent reproductive health services than those with low levels of knowledge after controlling for the variables of family and peer support.

The students with lower levels of knowledge tended to not utilize the services because of a lack of information about the available services. Thus, regular guidance on socialization with respect to reproductive health and available services could be provided through online media to increase students’ knowledge levels.

In this study, family support was determined to be a confounding variable with respect to the association between knowledge and the utilization of adolescent reproductive health services. More specifically, family support can affect students’ knowledge of adolescent reproductive health and available services because the family is one of the key sources of such information. In their questionnaires, some students admitted that they obtained information about reproductive health issues and services from their families. The existence of such support has a positive impact on knowledge and the utilization of adolescent reproductive health services. Therefore, family support needs to be improved by providing socialization programs for parents of adolescents and encouraging them to be more open to discussing their children’s reproductive health issues. Such information can be distributed with report cards at sessions attended by parents.

In addition to online media and family, peers are also a source of health information. This is one reason why peer support can influence the knowledge levels of students while also encouraging students to utilize the available services. Thus, peer support in this study was a confounding variable with respect to the link between knowledge and the use of adolescent health services. In everyday life, most teenagers spend time in school with others their age, so peers who become cadres/peer educators can further encourage the utilization the adolescent services. Three of the six schools in which the present research was conducted had organizations or extracurricular activities pertaining to adolescent reproductive health. One organization was the Center of Information and Counseling (PIK-R/M) under BKKBN, another was PERA (Anti-Drug Adolescent Association) and the thirds wasGANAS (National Anti-Drug Movement). Formed by the BNN, which designate students as cadres to disseminate information about reproductive health. Training adolescents to be peer educators and providing organizations or extracurricular activities geared toward informing young people about their reproductive health, can be a means of improving the utilization of health services.

Another means of encouraging the use of the services is individual perception. Interestingly, in the study, the four different perceptions, perceived susceptibility, severity and seriousness, benefits, or barriers, were found to be unrelated to the utilization of those services.

Both students with high and low perceived susceptibility tended to not utilize the services available. This result does not align with the findings of an Ethiopian study assessing reproductive health service utilization and perceptions among the same age group [24]. A possible reason for this different result is that the students in this study had lower levels of knowledge regarding reproductive health and available services, so it appears that there are still many students who are unaware of the existence or location of such services. Ethiopian adolescents experienced sex as a normal part of their life and they were open to discuss it with their mother [24]. Contrarily, in Indonesia disscussing about that topic is still taboo. It can be a reason that ehtiopian adolescent had higher knowledge about reproductive health and available services than Indonesian adolescents.

In terms of perceived severity and seriousness, that factor also appeared unrelated to the utilization of adolescent reproductive health services, a finding that once again contrasts with the Ethiopian study. The latter mentioned that adolescents with high perceived severity and seriousness are twice as likely to utilize adolescent reproductive health services [13]. In the current study, this was not found to be the case. Rather, students felt that if they had never engaged in risky behavior that could raise the likelihood of contracting a disease or other reproductive health issue(s), then there was no need to take advantage of adolescent reproductive health services.

Most of the students perceived benefits in relation to the utilization of adolescent reproductive health services. However, perceived benefits were not a factor affecting the actual utilization of adolescent reproductive health services. This is in line with the research conducted in Ethiopia [13]. One possibility leading to low utilization even with high-benefit perception, may be that the students believe they’re healthy so long as they show no unusual signs or symptoms.

Perceived barriers were also considered when looking at the utilization of health services. Previous research in Nepal found that the many barriers encountered were the cause of individuals not using the services [15]. The results of this study are not in line with those conclusions because the perceived barriers here were not related to the utilization of adolescent health services. One possible explanation may be the notion that reproductive health services are unnecessary or are not considered as important to students.

## Conclusion

The utilization of adolescent reproductive health services by high school students in Makassar City in 2018 was still relatively low (24.3%). Knowledge of available services was a driving factor in positively influencing their utilization rates. Well-informed students were nearly twice as likely to utilize adolescent reproductive health services after controlling for both family and peer support. The four perceptions of students (susceptibility, severity and seriousness, benefits, or barriers) were not found to be factors related to the utilization of adolescent reproductive health services.

Reproductive health service providers should provide means of socialization for students and parents on a regular basis, disseminate information through online media/social networks, and conduct peer educator training for each school. In addition, partnerships among the Health Department, Education Department, and other health institutions could be formed for the development of school organizations focusing on reproductive health. Future researchers are advised to conduct similar study by exploring variables such as social stigma and risky behavioral history as well as using qualitative methods to gain deeper insights into this critically important issue.

## Abbreviations

ARFHS: Adolescent Friendly Reproductive Health Service
BKKBN: National Family Planning Coordinating Board
BNN: National Narcotics Agency
GANAS: National Anti-Drug Movement
HBM: Health Belief Model
ICPD: International Conference on Population and Development
PERA: Anti-Drug Adolescent Association
PIK-R/M: Adolescent/Student Information and Counseling Center
PKPR: Adolescent Care Health Service
Sakernas: National Labor Force Survey
SMAN: Public Senior High School
